# Pulmonary nocardiosis mimicking lung cancer

**DOI:** 10.1590/0037-8682-0305-2026

**Published:** 2026-10-16

**Authors:** Alexandre Dias Mançano, Gláucia Zanetti, Bruno Hochhegger, Edson Marchiori

**Affiliations:** 1 University of Florida, Department of Radiology, Gainesville, FL, USA.; 2 Universidade Federal do Rio de Janeiro, Departamento de Radiologia, Rio de Janeiro, RJ, Brasil.

A 68-year-old immunocompetent, non-smoking woman developed a persistent 3-week cough, accompanied by chest pain, dyspnea, hemoptysis, fever, weight loss, and night sweats. Laboratory test results were unremarkable. Chest computed tomography (CT) revealed a mass with a necrotic focus in the right upper pulmonary lobe (Figure 1A-D ). Positron emission tomography/CT showed high fluorodeoxyglucose uptake in the mass and in the ipsilateral hilar lymph nodes (Figure 1E). These findings were considered suspicious for neoplastic disease. Brain magnetic resonance imaging, performed for staging, revealed a 1.2-cm lesion in the right frontal cortex with peripheral enhancement and mild perilesional edema, suggestive of metastasis (Figure 2). The patient underwent bronchoscopy followed by a percutaneous lung biopsy. Histopathological findings were negative for malignancy. Cultures and microbiological studies of bronchoalveolar lavage identified *Nocardia niwae.* The final diagnosis was cerebral and pulmonary nocardiosis (PN). The patient received intravenous imipenem and trimethoprim-sulfamethoxazole, resulting in significant clinical improvement and near-complete resolution of the pulmonary (Figure 3) and cerebral lesions. Treatment for at least 12 months was planned.

**FIGURE 1:**
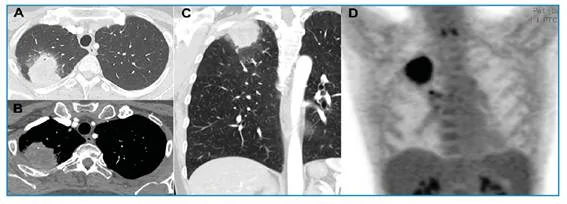
Chest computed tomography (CT) images with the axial lung **(A)**, mediastinal **(B)** windows, and coronal reconstruction **(C)** showing a heterogeneous mass with surrounding ground-glass opacities in the right upper pulmonary lobe. **(D)** Positron emission tomography/CT image showing high fluorodeoxyglucose uptake of the mass and ipsilateral hilar lymph nodes.

**FIGURE 2:**
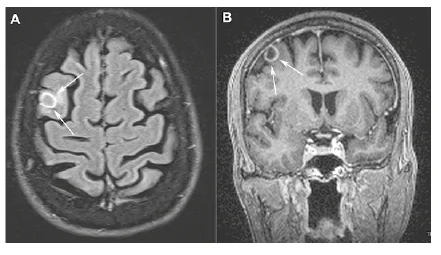
Axial T2 fluid-attenuated inversion recovery **(A)** and coronal T1 **(B)** post-contrast magnetic resonance images of the brain, showing a peripherally enhancing cortical lesion in the right frontal lobe with mild perilesional edema (arrows).

**FIGURE 3:**
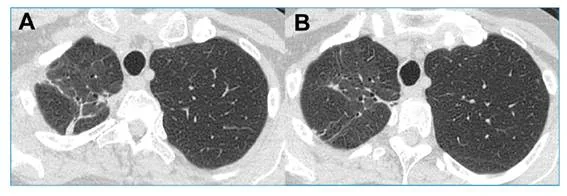
(A and B) Chest computed tomography images with the axial lung window, acquired 4 months after the start of treatment, showing reabsorption of the mass in the right upper lobe, with only slight residual band opacities.

PN is an opportunistic infectious disease caused by *Nocardia* species. Most Nocardia infections occur in individuals with compromised immune function. However, some *Nocardia* species also infect immunocompetent individuals. Diagnosis is particularly challenging in these individuals because symptoms and imaging findings are nonspecific.^1^ ^-^ ^5^ Common CT findings include lung consolidation, nodules, and masses with or without cavitation. *Nocardia* infection can thus mimic other infections, and even lung cancer. Definitive diagnosis requires microbiological confirmation^2^ ^-^ ^5^.
